# A centralized hybrid model for regulatory inspection readiness in a multi‐site radiation oncology system

**DOI:** 10.1002/acm2.70791

**Published:** 2026-09-10

**Authors:** Chaitanya Kalavagunta, Dustin Simonson, Mariana Guerrero

**Affiliations:** ^1^ Department of Radiation Oncology University of Maryland School of Medicine Baltimore Maryland USA; ^2^ Frederick Health Radiation Medicine Frederick Maryland USA

**Keywords:** Linac quality assurance, Multi‐site, Radiation oncology workflow, Regulatory inspection

## Abstract

**Background:**

State regulatory inspections of radiation‐producing treatment equipment combine a review of quality assurance and regulatory records with direct verification of facility and safety systems. When these activities are performed entirely on‐site across multiple facilities, real‐time document retrieval can prolong visits and divert clinical staff.

**Purpose:**

To develop and evaluate a centralized hybrid framework that separates advance document review from focused on‐site verification during state‐mandated annual inspections of photon linear accelerators.

**Methods:**

The framework was implemented across five photon treatment sites comprising 10 linear accelerators using an institutionally deployed cloud‐based quality assurance platform (TotalQA, Image Owl Inc., Greenwich, NY). Site physicists uploaded machine‐ and site‐specific records directly. A dedicated guest account allowed the inspector to review documents approximately 14 days before each visit. Document‐verifiable items and photographs of required postings were reviewed remotely; audio/video monitoring systems, linac‐on warning lights, staff access to applicable state regulatory requirements and personnel dosimetry records, written operating and emergency procedures, direct questions, and unresolved items remained on‐site. A system‐wide clinical deviation logbook containing protected health information was reviewed once in person at a central location. Operational outcomes included on‐site duration, daily throughput, physicist support, schedule adherence, treatment impact, and inspection findings.

**Results:**

Historical inspections required approximately 120 min of on‐site time per facility, based on calendar records, prior schedules, and staff recollection. During the hybrid implementation, on‐site inspection time was approximately 30 minutes per facility based on records documented at the time of each inspection, representing an estimated 75% reduction. Daily throughput increased from approximately three sites per day to four sites per day. The inspector estimated that advance review required 1 to 2 h per site, generally closer to 2 h during the first cycle. All visits remained within scheduled blocks, no inspection‐related treatment delays or interruptions occurred, and no documentation‐related citations were identified.

**Conclusions:**

A centralized hybrid model was feasible for state‐mandated annual inspections of linear accelerators in a multi‐site radiation oncology system. Advance review reduced on‐site document‐navigation demands while preserving direct verification of physical and safety‐related elements. Because remote‐review time was estimated retrospectively, the findings support reduced on‐site duration rather than reduced total inspector effort.

## 1 INTRODUCTION

In the United States, radiation‐producing machines are primarily regulated through state radiation‐control programs. A regulatory inspection is a formal evaluation conducted by a state agency or state‐licensed radiation machine inspector to determine whether a facility, its radiation‐producing equipment, and its radiation‐safety program comply with applicable registration, certification, radiation protection, and operating requirements. Inspections may be periodic, associated with initial operation, relocation, or decommissioning; required after replacement or substantial rebuilding of a major component; or conducted on an unscheduled basis in response to complaints, incidents, or other regulatory concerns. Activities may include review of regulatory forms, machine quality assurance and calibration records, personnel monitoring, required postings, operating and emergency procedures, and direct observation or functional verification of treatment‐room safety systems. These inspections are closely linked to broader quality assurance programs established within radiation oncology physics practice.^1^, ^2^, ^3^, ^4^

Traditional inspection workflows combine documentation review and physical verification into a single on‐site process. As a result, inspectors must locate, interpret, and validate documentation during the visit, increasing both inspection duration and disruption to clinical operations. These challenges are amplified in consolidated systems with multiple facilities and standardized equipment configurations.

Radiation oncology programs may maintain quality assurance and regulatory records in centralized electronic repositories, including institutional network drives, Microsoft SharePoint or OneDrive, enterprise document‐management systems, cloud‐based storage platforms, and dedicated quality assurance software. In this context, a centralized document management system refers to a controlled repository in which records from multiple facilities are organized using standardized site, machine, document‐type, and date identifiers and are accessible to authorized users through defined permissions. Such systems can support consistent organization, document retrieval, version control, and record sharing across sites. Published reports of centralized and automated QA management systems have demonstrated improved QA completion compliance and support for standardized QA management, monitoring, and data retrieval.^5^, ^6^ However, their use as infrastructure for state regulatory inspection readiness has received less attention, particularly regarding how inspection activities can be divided between advance document review and on‐site verification while maintaining compliance and safety. Published remote‐inspection experience supports the use of document review to complement, rather than replace, direct observation and functional verification.^7^, ^8^

This technical note describes a centralized hybrid model used during annual state‐mandated inspections of photon linear accelerators across five treatment sites. The model separates advance documentation review from focused on‐site verification. The evaluated implementation was limited to external‐beam linear accelerators and did not include computed tomography simulators, brachytherapy, proton therapy, or patient‐chart review. The framework was designed to support asynchronous review of inspection documentation, standardize document organization and access, and reduce on‐site inspection time while preserving direct verification of safety‐critical elements. This note describes the model's implementation and operational performance within a multi‐site radiation oncology system.

## 2 METHODS

### 2.1 System setting and scope

The framework was implemented during the first inspection cycle of state‐mandated annual radiation‐machine inspections across five photon treatment sites comprising ten linacs: three at Site 1, two each at Sites 2–4, and one at Site 5. The inspection was conducted by a state‐licensed radiation machine inspector. Computed tomography simulators, brachytherapy systems, proton therapy systems, and patient‐chart review were outside the evaluated scope.

### 2.2 Regulatory mapping framework

Inspection requirements were categorized according to the nature and location of verification. Four primary categories were defined:

- **Machine‐specific documentation** (e.g., TG‐51 calibration reports,^1^ periodic QA documentation,^3^, ^4^ preventative maintenance inspection [PMI] reports, IROC output checks)
- **Site‐ and system‐specific documentation** (e.g., regulatory forms, licensure, registration, personnel dosimetry records, operating and emergency procedures, and photographs of required postings)
- **Physical and operational verification elements** (e.g., audio/video monitoring systems, linac‐on warning lights, staff access to applicable state regulatory requirements and personnel dosimetry records, and the treatment‐area tour)
- **Sensitive system‐level documentation requiring controlled in‐person review** (the clinical deviation logbook containing protected health information and other sensitive content)

Document‐verifiable records were assigned to advance review, whereas elements requiring direct observation, functional testing, confirmation of local staff access, or controlled review because of privacy considerations remained for in‐person review. The inspector retained authority over the final inspection scope. A risk‐informed mapping approach was used to organize the workflow (Table **1**); however, the inspector retained full professional discretion to request additional documentation, perform direct verification, or expand the on‐site review as needed.

**TABLE 1:** Risk‐informed assignment of inspection elements to advance remote, centralized in‐person or on‐site review pathways. The inspector retained authority over the final inspection scope; the mapping was used to organize document‐verifiable, physically verifiable, and privacy‐sensitive elements.

Inspection element	Risk level	Purpose	Review modality
TG‐51 Calibration	High	Absolute dose accuracy	Remote
PMI Reports	High	Mechanical/electrical performance	Remote
IROC Output Checks	High	Independent dose verification	Remote
QA Records (Daily/Monthly/Annual)	Moderate	Routine performance and trend review	Remote
Personnel Dosimetry Records	High	Occupational radiation exposure compliance	Remote
Regulatory Forms, Licensure, and Registration	Moderate	Regulatory compliance	Remote
Photographs of Current Registration and Notice to Employees	Moderate	Posting presence and location	Remote
Clinical Deviation Logbook	High	Incident and corrective‐action review	Centralized in‐person
Audio/Video Monitoring Systems	High	Patient monitoring safety	On‐site
Linac‐On Warning Lights	High	Radiation safety indication	On‐site
Staff Access to Applicable State Regulatory Requirements and Personnel Dosimetry Records	Moderate	Availability of required information	On‐site
Written Operating and Emergency Procedures	High	Operational and emergency preparedness	On‐site
Treatment‐Area Tour, Direct Questions, and Outstanding Items	Moderate	Physical context and clarification	On‐site

### 2.3 Centralized platform and document submission

TotalQA (Image Owl Inc., Greenwich, NY), a cloud‐based QA platform already deployed across the institution, served as the centralized repository. It was selected because all participating physicists already had access, records could be associated with specific sites and linacs, and a guest account could be created for remote access by the inspector. No formal comparison with alternative software platforms was performed.

### 2.4 Documentation taxonomy and access model

A standardized documentation taxonomy and access model was developed to support remote inspection (Table 2). Each site physicist uploaded applicable site‐ and machine‐specific documents directly into TotalQA. Once uploaded, documents were immediately accessible to the inspector through the assigned guest account, eliminating the need for a separate document‐transfer step. Documents were organized using consistent metadata tagging, including:

- Site identifier
- Machine identifier
- Document type
- Inspection year

**TABLE 2:** Documentation taxonomy organized by review modality, scope, category, and examples.

Review modality	Scope	Category	Examples
Remote	Linac	QA, calibration, and maintenance	TG‐51; daily/monthly/annual QA; PMI; IROC output checks
Remote	Site/system	Regulatory and administrative	Inspection forms; licenses; registration; personnel dosimetry records; photographs of required postings
Remote (conditional)	Linac	Acceptance and commissioning	Initial inspection, major equipment change, or specific inspector request
Centralized in‐person	System	Sensitive records	Clinical deviation logbook
On‐site	Site/linac	Safety and operations	Audio/video monitoring systems; linac‐on warning lights; staff access to applicable state regulatory requirements and personnel dosimetry records; written operating and emergency procedures; treatment‐area tour; direct questions; outstanding items

A dedicated guest account was created using the inspector's email address, and the platform sent an automated new‐user message containing the access link. Written instructions explained how to filter records by site, machine, and document type and provided a link to the platform's document‐navigation guidance. Access was configured for document review rather than modification of records, and the inspector was asked not to download documents. No real‐time navigation assistance was required during advance review.

### 2.5 Data governance protocol

A structured document‐control and access framework was applied to define document ownership and accessibility and ensure consistency (Table 3). Key elements included:

- Version control and identification of the current approved document
- Mandatory metadata tagging
- Linking machine‐specific reports to corresponding linac records
- Standardized navigation pathways for inspector access

**TABLE 3:** Document‐control and access framework supporting consistent organization, controlled inspector access, and independent document retrieval across multiple facilities.

Framework element	Implementation standard	Purpose
Version control	Current inspection documents identified consistently	Ensures consistency and traceability
Metadata tagging	Site, machine, document type, year	Enables structured filtering
Document linkage	Reports linked to specific linacs or sites	Reduces ambiguity
Naming convention	Standardized file naming	Improves searchability
Access control	Dedicated guest account configured for document review	Supports controlled inspector access
Navigation structure	Filter‐based document retrieval with written guidance	Enables independent review

This structure enabled the inspector to locate required documents independently. Site physicists remained responsible for the completeness and accuracy of records uploaded for their sites, while a designated central medical physicist established the taxonomy, coordinated access, and served as the point of contact for technical questions.

### 2.6 Inspection workflow design

**FIGURE 1:**
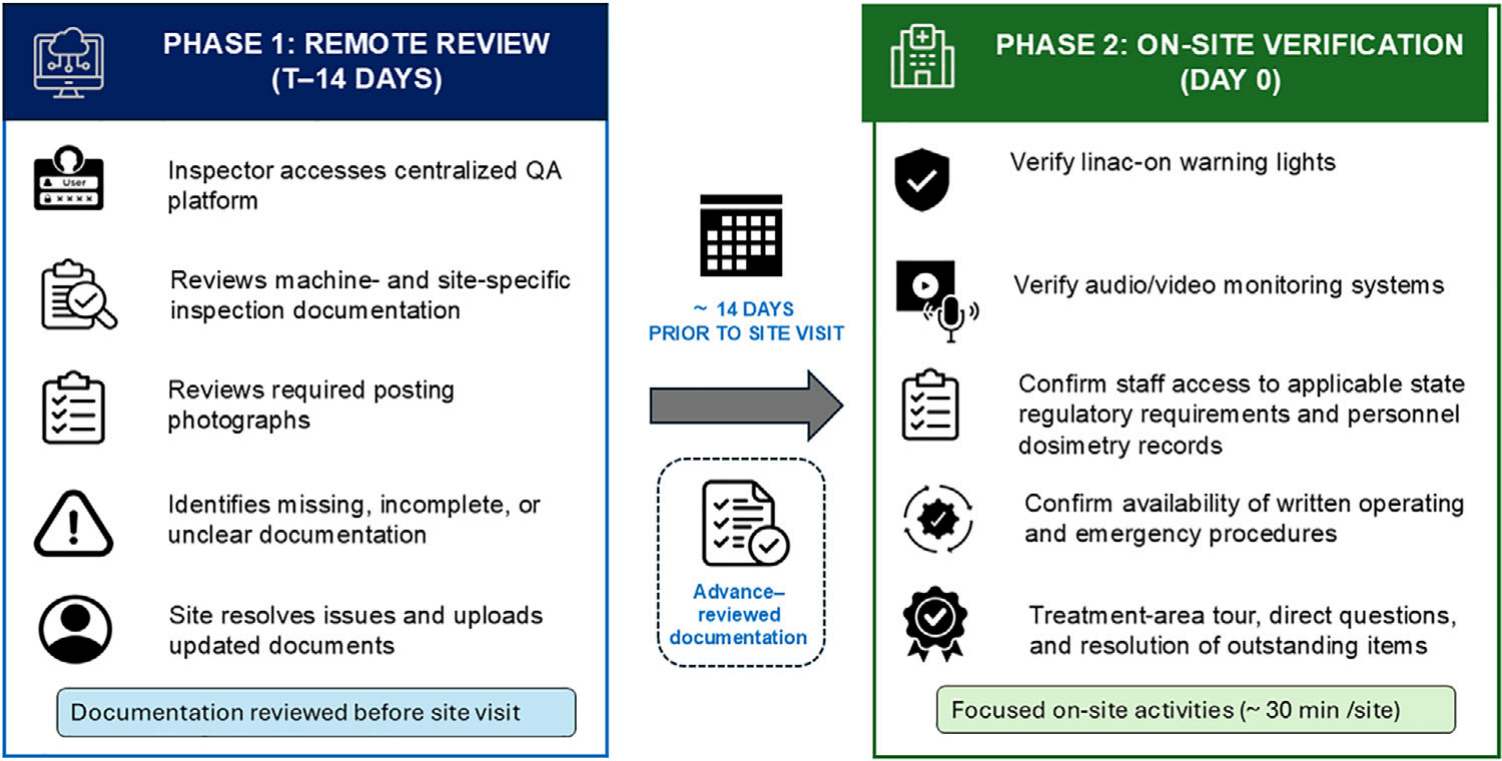
**Two‐phase hybrid inspection model illustrating separation of advance remote review and on‐site verification**. Inspection activities were divided into a remote‐review phase beginning approximately 14 days before the site visit and a focused on‐site phase on Day 0. Sensitive system‐level records that were not suitable for cloud access were reviewed once in person at a central location.

The inspection process was divided into an advance remote‐review phase and a focused on‐site verification phase (Figure 1). Sensitive system‐level records unsuitable for cloud access were reviewed once in person at a central location.

### 2.7 Advance remote review phase (T‐14 days)

The inspector performed an asynchronous review approximately 14 days before the site visits. Missing, incomplete, or unclear records were communicated by email to the applicable site physicist and corrected before the on‐site inspection. The inspector retrospectively estimated that remote review required approximately 1 to 2 h per site and was generally closer to 2 h during the first cycle while becoming familiar with the platform.

### 2.8 On‐site verification phase (Day 0)

The on‐site phase focused on elements requiring direct observation, functional verification or confirmation of staff access:

- Verification of audio and video monitoring systems and linac‐on warning lights
- Confirmation that staff had access to applicable state regulatory requirements and personnel dosimetry records and that written operating and emergency procedures were available
- Treatment‐area tour, direct questions, and resolution of outstanding items

Photographs showing the posted current facility registration and Notice to Employees were uploaded to TotalQA and reviewed during the advance phase; therefore, these postings were not rechecked on‐site. The system‐wide clinical deviation logbook was not uploaded because it contained protected health information and other sensitive content and was instead reviewed once in person at a central location. The inspector retained discretion to request additional records or verification as needed.

### 2.9 Operational Metrics and Evaluation

Operational performance was assessed using the following metrics:

- On‐site inspection duration per site
- Daily inspection throughput
- Schedule adherence
- Clinical workflow impact (site physicist support and treatment operations)
- Compliance outcomes (inspection citations)

Historical on‐site duration under the traditional workflow was reconstructed retrospectively from calendar records, prior inspection schedules, and staff recollection and was therefore treated as an approximate internal estimate. On‐site duration during the hybrid workflow was determined from inspector schedules, recorded arrival and departure times, calendar blocks, direct observation, and emails from site physicists sent during or shortly after the inspection. Because a site physicist actively supporting the inspector is temporarily unavailable for concurrent clinical responsibilities, clinical workflow impact was evaluated separately from treatment interruption. Appropriate physics coverage should therefore be available during a scheduled inspection. Moderate clinical disruption was defined as repeated physicist involvement totaling approximately 2 to 3 h to retrieve or explain records; minimal disruption was defined as approximately 30 min or less of active involvement, primarily for direct questions and the site tour. Treatment interruption was evaluated separately as an inspection‐related delay, cancellation, or suspension of scheduled treatment.

### 2.10 Implementation effort

Initial implementation required structured organization and direct upload of inspection documentation by site physicists, including metadata tagging and validation of document completeness. Ongoing maintenance involved periodic record updates and verification of version control. Supplementary Material S1 provides a practical implementation checklist based on the evaluated workflow, including pre‐implementation feasibility assessment, document inventory and pathway assignment, repository configuration, controlled inspector access, internal pre‐review, issue resolution, on‐site preparation, post‐inspection follow‐up, and ongoing inspection readiness. The checklist is intended for adaptation to local regulatory requirements, institutional policies, and available digital infrastructure.

### 2.11 Use of artificial intelligence tools

Artificial intelligence‐based language tools (ChatGPT, OpenAI, San Francisco, CA) were used to assist with manuscript editing, language refinement, organizational restructuring, and conceptual development of graphical layouts. All technical content, workflow design, operational analysis, figures, and final manuscript review were performed and verified by the authors. The authors assume full responsibility for the content of the manuscript.

## 3 RESULTS

The hybrid workflow was implemented across all five sites and ten linacs. Retrospective reconstruction from prior calendars, inspection schedules, and staff recollection indicated that traditional annual inspections required approximately 120 min of on‐site time per facility. During the hybrid implementation, on‐site duration documented at the time of each inspection was approximately 30 min per facility, representing an estimated 75% reduction in on‐site time (Table 4).

**TABLE 4:** Descriptive comparison of the historical traditional workflow and the hybrid implementation. Traditional‐model values were retrospectively reconstructed; hybrid on‐site duration was documented at the time of each inspection, whereas remote‐review time was retrospectively estimated by the inspector.

Metric	Traditional model	Hybrid model	Interpretation
On‐site inspection time	∼120 min/site; retrospective estimate	∼30 min/site; documented at time of inspection	∼75% lower on‐site duration
Daily throughput	∼3 sites/day	4 sites/day	One additional site completed per day
Site physicist support	Repeated support over ∼2‐3 h	Direct questions and site tour; generally ≤ 30 min	Moderate versus minimal clinical disruption
Advance inspector review	Not separated from on‐site visit	Estimated 1 to 2 h/site; closer to 2 h initially	Total inspector time not prospectively compared
Clinical coverage and treatment operations	Physicist occupied with repeated inspection support; coverage required for concurrent clinical duties	Limited physicist support; no treatment delay, cancellation, or interruption	Physicist availability and treatment continuity evaluated separately

The inspector estimated that advance document review required approximately 1 to 2 h per site and was generally closer to 2 h during the first cycle. Findings from the remote review and subsequent follow‐up were documented through email correspondence. Because this time was not prospectively logged, total inspector effort was not compared between workflows.

Daily inspection throughput increased from approximately three to four sites per day. Site physicist involvement during the hybrid visits consisted primarily of direct questions and the treatment‐area tour and generally required approximately 30 min per site.

Advance review identified missing or incomplete documentation in a subset of inspections, allowing corrective action before the on‐site visits. As a result, no documentation deficiencies required resolution through prolonged on‐site document retrieval.

All visits were scheduled in advance and completed within predefined scheduling blocks. No inspection‐related treatment delay, cancellation, or interruption occurred, and no documentation‐related citations were identified. The inspector reported that remote access supported a more orderly review at a convenient time without disrupting clinical staff or the inspection schedule; navigation required brief initial familiarization but became straightforward to use, and no assistance was required during remote review.

## 4 DISCUSSION

This first‐cycle implementation demonstrates the feasibility of separating annual state inspection activities into advance document review and focused on‐site verification across a multi‐site photon treatment network. The principal observed benefit was an estimated reduction in on‐site duration and site‐physicist support, not a demonstrated reduction in total inspector effort.

Even when an inspection is scheduled in advance, a physicist actively assisting the inspector is temporarily unavailable for other clinical responsibilities. Sites should therefore ensure appropriate physics coverage during the inspection period. Reducing repeated document‐navigation support may preserve physicist availability for concurrent clinical work, although this study did not formally measure effects on other clinical tasks.

The risk‐informed mapping approach provided a structured way to distinguish records suitable for asynchronous review from activities requiring direct observation, functional verification, confirmation of staff access, or controlled in‐person review. In the evaluated inspections, audio/video monitoring systems, linac‐on warning lights, staff access to applicable state regulatory requirements and personnel dosimetry records, written operating and emergency procedures, direct questions, and the treatment‐area tour remained on‐site. Photographs of the current registration and Notice to Employees were reviewed remotely and not rechecked during the visit.

The workflow depended less on the specific commercial platform than on the organizational structure applied within it. A common taxonomy, clear ownership of records, machine‐level linkage, and controlled inspector access are the transferable elements. This interpretation is consistent with reports that centralized QA databases improve standardization, record retrieval, and completion monitoring in large radiation oncology networks.^5^, ^6^

The hybrid design also aligns with published experience showing that remote regulatory methods are well suited to procedures, documents, and records but are less able to replace direct observation and functional verification.^7^, ^8^ The inspector in this implementation found the remote review useful and orderly and required no navigation assistance after brief familiarization, supporting broader adoption where permitted by regulatory policy.

Successful implementation of the hybrid model requires both inspector willingness to conduct advance remote review and a regulatory framework that permits remote access to inspection records. Unannounced inspections may not permit a formal advance‐review interval, although a continuously maintained centralized repository could still improve readiness and rapid record retrieval. The model also accommodated records that were unsuitable for remote access: the system‐wide clinical deviation logbook was reviewed once at a central location rather than uploaded or presented repeatedly at each site.

Patient‐chart review was outside the scope of these annual machine inspections. A broader hybrid framework could incorporate risk‐based selection of charts representative of the treatment techniques performed at each site, but that extension was not evaluated here. Future efforts will focus on extending this model to include brachytherapy, computed tomography simulation, and proton therapy systems.

## 5 LIMITATIONS

Because this was a descriptive, single‐system, first‐cycle implementation involving one regulatory inspection context and one inspector, the findings should be interpreted as operational feasibility rather than proof of superiority or long‐term performance. The historical comparison was reconstructed retrospectively, and the inspector's remote‐review time was estimated rather than prospectively recorded.

Additional cycles are needed to assess reproducibility across inspectors, regulatory programs, equipment types, and time periods. The model depends on consistent document‐control practices, secure access, and inspector willingness to perform advance review. Certain elements remain inherently site‐dependent, and the findings should not be generalized to inspections that include patient‐chart review, computed tomography simulators, brachytherapy, proton therapy, or other modalities not evaluated here.

## 6 CONCLUSION

A centralized hybrid model was feasible for state‐mandated annual inspections of 10 photon linear accelerators across five treatment sites. Advance review of standardized records reduced the need for on‐site document navigation while preserving direct verification of physical, operational, and safety‐related elements. These findings support broader evaluation of the model but do not establish reduced total inspector effort.

## AUTHOR CONTRIBUTIONS

All three authors conceived and developed the hybrid inspection framework. Chaitanya Kalavagunta implemented the inspection framework in TotalQA, performed the workflow and operational analysis, prepared the figures and tables, and drafted the manuscript. Mariana Guerrero and Dustin Simonson provided manuscript review, editorial feedback, and approval of the final submitted version.

## CONFLICT OF INTEREST STATEMENT

The authors have no relevant conflicts of interest to disclose.

## ETHICS STATEMENT

Not applicable. This work did not involve human participants, collection or analysis of identifiable patient data, or animal research and therefore did not require institutional review board approval.

## Supporting information




**Supporting Information**: acm270791‐sup‐0001‐SuppMat.docx

## Data Availability

The data supporting the findings of this study are included within the article and its supplementary material. Additional information is available from the corresponding author upon reasonable request.

## References

[1] Almond PR , Biggs PJ , Coursey BM , et al. AAPM's TG‐51 protocol for clinical reference dosimetry of high‐energy photon and electron beams. Med Phys. 1999;26(9):1847‐1870. 10505874 10.1118/1.598691

[2] Huq MS , Fraass BA , Dunscombe PB , et al. The report of task group 100 of the AAPM: application of risk analysis methods to radiation therapy quality management. Med Phys. 2016;43(7):4209‐4262. 27370140 10.1118/1.4947547 PMC4985013

[3] Klein EE , Hanley J , Bayouth J , et al. Task group 142 report: quality assurance of medical accelerators. Med Phys. 2009;36(9):4197‐4212. 19810494 10.1118/1.3190392

[4] Krauss RF , Balik S , Cirino ET , et al. AAPM medical physics practice guideline 8.b: linear accelerator performance tests. J Appl Clin Med Phys. 2023;24(11):e14160. 37793084 10.1002/acm2.14160 PMC10647991

[5] Lyatskaya Y , Killoran J , Kukluk J , Cormack R , and Quirk S . Improving management and compliance of radiation therapy linear accelerator quality assurance program with automated tracking tools. Adv Radiat Oncol. 2024;9(5):101469. 38550367 10.1016/j.adro.2024.101469 PMC10972807

[6] Tang G , LoSasso T , Chan M , Hunt M . Impact of a centralized database system on radiation therapy quality assurance management at a large health care network: 5 years’ experience. Pract Radiat Oncol. 2022;12(5):e434‐e441. 35431152 10.1016/j.prro.2022.03.003 PMC9452445

[7] Hourdakis CJ , Vogiatzi S , Pafilis C , et al. Remote virtual regulatory inspections of facilities and practices utilising ionising radiation and MRI installations during the Covid‐19 pandemic. J Radiol Prot. 2021;41(4):1184. 10.1088/1361-6498/ac31c2 34673558

[8] Mofid S , Bolislis WR , Brading C , et al. The utility of remote inspections during the COVID‐19 health emergency and in the postpandemic setting. Clin Ther. 2021;43(12):2046‐2063. 34740466 10.1016/j.clinthera.2021.10.003 PMC8511654

