# Supplementary material for: A centralized hybrid model for regulatory inspection readiness in a multi‐site radiation oncology system
Source: J Appl Clin Med Phys. 2026 Sep 10;27(9):e70791. doi: 10.1002/acm2.70791 (PMC13559466; doi:10.1002/acm2.70791)
Supplement: Supplementary file 1 [file ACM2-27-e70791-s001.docx]

## Supplementary Material S1

### Implementation Checklist for Centralized Hybrid Inspection Model

**Purpose:** This checklist provides a practical framework for implementing a centralized hybrid regulatory inspection workflow in a multi-site radiation oncology system. The model combines advance remote document review with focused on-site verification while preserving the inspector’s authority to expand the review as needed.

The checklist should be adapted to applicable regulatory requirements, institutional policies, inspection practices, and available digital infrastructure.

#### **Step 1 — Confirm Feasibility and Regulatory Alignment**

☐ Confirm that the regulatory authority and assigned inspector are willing to conduct advance remote document review.

☐ Determine whether the inspection is scheduled or may occur without advance notice.

☐ Confirm which records may be reviewed remotely and which must remain available on-site.

☐ Identify institutional privacy, cybersecurity, records-management, and vendor requirements.

☐ Establish an institutional point of contact for coordinating the inspection.

#### **Step 2 — Develop the Inspection Document Inventory**

Identify all records that may be required for inspection, including applicable:

**Machine-specific records**

☐ Reference dosimetry and calibration reports, including TG-51 documentation
☐ Daily, weekly, monthly, and annual QA records
☐ Preventive maintenance and service records
☐ Independent output-verification results, such as IROC reports
☐ Equipment calibration certificates
☐ Machine-specific corrective-action records

☐ Acceptance and commissioning records, when required for an initial inspection, major equipment change, or specific inspector request

**Site-specific records**

☐ Facility registration records
☐ Personnel dosimetry records
☐ Radiation safety training records
☐ Physician licenses
☐ Radiation therapist licenses
☐ Required notices and posting photographs
☐ Written operating procedures
☐ Written emergency procedures
☐ Applicable regulatory documents available to staff

**System-level or sensitive records**

☐ Clinical deviation or event logbook
☐ Records containing protected health information or other sensitive information
☐ Documents maintained centrally rather than separately at each facility

#### **Step 3 — Assign Inspection Elements to Review Pathways**

Classify each inspection element according to how it should be reviewed.

**Pathway A: Advance remote review**

Use for records that can be evaluated from documentation, such as:

☐ QA reports
☐ Calibration and dosimetry records
☐ Preventive maintenance records
☐ Facility registration records
☐ Personnel records
☐ Training records
☐ Required posting photographs

**Pathway B: On-site verification**

Use for elements that must be physically observed, tested, or discussed, such as:

☐ Treatment-area walkthrough
☐ Required signage or postings when direct verification is required or advance photographic review is not accepted
☐ X-ray warning lights
☐ Linac-on indicators
☐ Door and safety interlocks
☐ Audio and video monitoring systems
☐ Availability of operating and emergency procedures
☐ Staff access to applicable regulations and dosimetry information
☐ Staff questions
☐ Resolution of outstanding inspection items

**Pathway C: Centralized in-person review**

Coordinate one controlled centralized in-person review of designated system-level sensitive records rather than uploading or duplicating them at each site. Use for sensitive or system-level records that should not be uploaded or duplicated across sites, such as:

☐ Clinical deviation or event logbooks
☐ Records containing protected health information
☐ Other centrally maintained confidential records

#### **Step 4 — Establish a Standardized Repository Structure**

☐ Create a centralized location for inspection-related documentation.
☐ Organize records using a consistent hierarchy, such as:

- Site
- Treatment machine
- Document type
- Inspection year

☐ Link machine-specific records to the corresponding treatment unit.
☐ Separate site-specific records from machine-specific records.
☐ Clearly identify records maintained centrally or reviewed only on-site.

**Step 5 — Standardize Metadata and Naming Conventions**

☐ Define required metadata fields.
☐ Apply consistent tags for:

- Site
- Machine
- Document type
- Inspection year

☐ Develop a standardized file-naming convention.
☐ Include the document date or applicable year where appropriate.
☐ Use version control when records are updated.
☐ Remove obsolete or duplicate versions from the active inspection view.
☐ Confirm that the most current approved record is readily identifiable.

**Step 6 — Configure the Digital Platform**

☐ Upload the required records to the centralized repository.
☐ Configure filters for site, machine, document type, and year.
☐ Confirm that records can be located without assistance from individual site personnel.
☐ Validate version-control functionality.
☐ Confirm that access permissions are consistent with institutional policies.
☐ Test the platform from a non-administrator account.
☐ Verify that the repository can be accessed from outside the institutional network when permitted.

The model may be implemented using TotalQA or another secure repository capable of supporting standardized organization, controlled access, and document retrieval.

**Step 7 — Establish Inspector Access**

☐ Create a time-limited, role-restricted inspector account.
☐ Configure review-only access when supported.
☐ Restrict editing privileges.
☐ Restrict downloading when supported and required by institutional policy; otherwise, provide written instructions that documents are for review only and should not be downloaded.
☐ Confirm that the inspector can view the necessary sites, machines, and document categories.
☐ Provide concise written navigation instructions.
☐ Include contact information for access or technical problems.
☐ Deactivate the account after the inspection when appropriate.

**Step 8 — Conduct Internal Pre-Review**

Before providing inspector access:

☐ Confirm that all required records are present.
☐ Verify that documents are assigned to the correct site and machine.
☐ Confirm that document titles, metadata, and dates are accurate.
☐ Check that required signatures or approvals are present.
☐ Confirm that expired or superseded records are clearly identified.
☐ Verify that sensitive information has not been uploaded unintentionally.
☐ Test filters and navigation from the inspector’s perspective.
☐ Document any records that will be shown centrally or on-site rather than uploaded.

**Step 9 — Coordinate Advance Remote Review**

For a scheduled inspection:

☐ Provide access approximately 14 days before the on-site visit, or according to the agreed schedule.
☐ Notify the inspector that the repository is ready for review.
☐ Provide navigation instructions and a summary of the repository structure.
☐ Establish a communication pathway for questions and missing-document requests.
☐ Ask the inspector to identify unresolved items before the site visit when feasible.
☐ Retain written documentation of remote-review questions and responses.

Advance remote review depends on inspector participation and may not be feasible for unannounced inspections or where regulatory policy requires all review to occur on-site.

**Step 10 — Resolve Issues Identified During Remote Review**

☐ Respond to inspector questions promptly.
☐ Upload missing records when appropriate.
☐ Correct incomplete or incorrectly categorized documentation.
☐ Add updated versions while maintaining version traceability.
☐ Document the resolution of each identified issue.
☐ Identify items that must remain open for on-site verification.
☐ Confirm repository completeness before the scheduled visit.

**Step 11 — Prepare for the On-Site Inspection**

☐ Develop a site-specific verification checklist.
☐ Confirm that treatment rooms and support areas are accessible.
☐ Verify operation of:

- Door and safety interlocks
- Audio monitoring systems
- Video monitoring systems
- X-ray warning lights
- Linac-on indicators

☐ Confirm required signs and postings on-site when they were not accepted through advance photographic review or when direct verification is requested by the inspector.
☐ Ensure that applicable regulations are accessible to staff.
☐ Ensure that personnel dosimetry information is available as required.
☐ Make written operating and emergency procedures available.
☐ Prepare non-digitized records and confirm availability of central-only records through their designated review pathway.
☐ Ensure that appropriate site personnel are available for questions.
☐ Prepare responses to any unresolved remote-review items.

**Step 12 — Conduct the On-Site Inspection**

☐ Provide a treatment-area and facility walkthrough.
☐ Support physical verification of safety-critical systems.
☐ Present non-digitized or other records designated for on-site review.
☐ Address staff and inspector questions.
☐ Resolve outstanding items from the remote review.
☐ Provide additional records when requested.

The hybrid model is intended to focus the on-site visit on activities requiring direct observation, testing, discussion, or clarification. It does not restrict the inspector’s judgment, authority, or ability to expand the inspection scope.

**Step 13 — Complete Post-Inspection Follow-Up**

☐ Document inspection findings and outcomes.
☐ Correct any deficiencies or open items.
☐ Upload revised records when appropriate.
☐ Deactivate or modify inspector access.
☐ Record the approximate time required for remote and on-site activities.
☐ Obtain feedback from the inspector and participating sites.
☐ Identify barriers, duplicative steps, and opportunities for standardization.
☐ Update the repository structure and implementation checklist before the next inspection cycle.

**Step 14 — Maintain Continuous Inspection Readiness**

☐ Incorporate repository maintenance into routine QA operations.
☐ Assign responsibility for each record category.
☐ Establish document-upload deadlines.
☐ Periodically review metadata accuracy and record completeness.
☐ Remove or archive superseded records.
☐ Audit inspector-account permissions.
☐ Review the system after regulatory or organizational changes.
☐ Evaluate adaptation of the model to additional modalities, such as CT simulation, brachytherapy, and proton therapy.

***Implementation Note***

*The centralized hybrid model redistributes inspection work rather than eliminating it. Document review is moved upstream, while on-site time is preserved for activities requiring physical verification, staff interaction, and inspector judgment. Successful implementation requires standardized document organization, defined institutional ownership, secure inspector access, and cooperation from the regulatory authority.*
